# Singular Instance of Vomiting of Fat and Blood

**Published:** 1826-07

**Authors:** 


### VOMITING OF FAT AND BLOOD.—PASQUALI.

In the *Annali Univers.* for January, there is related the case of a man, aged 75, who had always enjoyed good health with the exception of an attack of jaundice. He was in the habit of fasting sometimes for a whole day or two, and then eating, in excess, the most indigestible substances. This went on for many years, without any apparent ill consequences; but the day of reckoning at length arrived. For two years past he had been seized with periodical vomitings, every week or a fortnight, attended with complete loss of appetite for some days; when the stomach would again become restored. One evening lately he was seized with a more severe attack than usual, after great imprudence in diet, and vomited most abundantly. When the paroxysm was apparently over, a new phenomenon took place, and Dr. Pasquali was called in. The matters thrown up were no longer the ordinary contents and secretions of the stomach, but a mixture of pure blood and a kind of thick oil or melted fat. This process went on to a prodigious extent, and our author calculated that the patient threw up, in the course of 24 hours, the enormous quantity of thirty pounds or pints of this mixture. The man was reduced to the brink of the grave, and life was scarcely perceptible, when the orgasm ceased. And now a surprising change was perceived in the patient's body. He had been rather embonpoint before the attack; but his skin was now hanging in folds, and the whole of the adipose substance seemed to have disappeared from the belly and every part of the body where it had previously abounded. The orgasm over, the poor man was nourished every hour with light liquid food, and life was thus preserved. In twenty days he was restored to health, but still with an immense loss of adipose substance.

Incredible as this case may appear, we believe it to be a fact. We have seen the stomach and the absorbents play such parts in the animal economy as would surprise and startle those who had not witnessed such feats. The above case also illustrates another fact—namely, that however long the stomach may bear with patience the insult which we daily offer that important organ, yet the day of retribution must come at last; and is generally the more terrible in proportion as it has been longer delayed. A hurricane, as it were, takes place in the constitution, and purges it of its morbid causes, or ill-gotten accumulations. It is thus that we see people laid up annually with certain constitutional diseases, as gout, indigestion, &c. which reduce the human fabric to a certain point, when the phenomena gradually cease, and the patient is anxious to make up the reduction in flesh by every kind of nutriment which himself or the cook can devise. He rises again to a certain point in the scale of (apparent) health, and thinks himself most fortunate in thus gaining such an ascendancy. But at this moment a cog flies from the machinery, and a morbid spring, which had long been gradually pressed down, is suddenly set free, a recoil in the constitution takes place, the disease is developed, and runs its course as before. It is, perhaps, not very material whether we call this periodical accession a salutary process or the natural consequence or effect of a train of morbid causes accumulating in the constitution. In either way we view it, the event is inevitable. Or, if evitable, the escape is worse than the attack. Every person who has experienced these periodical storms of the constitution will acknowledge that the subsequent amelioration of function in the stomach and other organs of the body, as well as of the mind, is quite extraordinary—sufficient indeed to raise and support the idea of the preceding disease being a restorative or healthy process. But, on this subject, we shall soon have a fuller opportunity to enlarge.

